# ﻿Four new species of *Zeugodacus* Hendel (Diptera, Tephritidae, Dacinae, Dacini) and new records of dacines from India

**DOI:** 10.3897/zookeys.1188.114031

**Published:** 2024-01-03

**Authors:** Karamankodu Jacob David, Venkateshaiah Abhishek, Ningthoujam Kennedy, K. M. Ajaykumara, R. G. Gracy, Cheday Bhutia Hissay

**Affiliations:** 1 National Bureau of Agricultural Insect Resources, Bengaluru-560024, Karnataka, India National Bureau of Agricultural Insect Resources Bengaluru India; 2 Keladi Shivappa Nayaka University of Agricultural and Horticultural Sciences, Shivamogga, Karnataka, India Keladi Shivappa Nayaka University of Agricultural and Horticultural Sciences Shivamogga India; 3 College of Post-Graduate Studies in Agricultural Sciences, CAU (Imphal), Umiam-793103, Meghalaya, India College of Post-Graduate Studies in Agricultural Sciences Umiam India; 4 College of Horticulture and Forestry, CAU (Imphal), Pasighat-791102, Arunachal Pradesh, India College of Horticulture and Forestry Pasighat India

**Keywords:** Arunachal Pradesh, cue lure, dacines, fruit fly, Meghalaya, Shimla, zingerone

## Abstract

Four new species of *Zeugodacus* Hendel are described from India viz., *Zeugodacusmomordicae* David & Ajaykumara, **sp. nov.** from Arunachal Pradesh infesting male flower buds of *Momordicadioica*, *Zeugodacusnasivittatus* David & Abhishek, **sp. nov.** from Meghalaya, Zeugodacus (Sinodacus) sinuvittatus David & Abhishek, **sp. nov.** from Himachal Pradesh and Zeugodacus (Zeugodacus) umiam David & Kennedy, **sp. nov.** from Meghalaya. An illustrated key to all species of *Zeugodacus* from India is also included. Bactrocera (Parazeugodacus) abbreviata (Hardy) and Dacus (Mellesis) vijaysegarani Drew & Hancock are recorded for the first time from India.

## ﻿Introduction

*Zeugodacus* Hendel is a genus in tribe Dacini with 196 species recorded from the world (Doorenweerd et al. 2018) and thirty described species from India (David et al. 2017; David and Ramani 2019). They are characterised by the shallow emargination of sternite 5 in males, posterior lobe of surstylus 5–6× longer than anterior lobe, glans of phallus with patterned acrophallus. Fruit flies of genus *Zeugodacus* Hendel are economically important as several of them are pests of various horticultural crops. *Zeugodacus* was originally treated as a subgenus of *Bactrocera* Macquart, it was elevated to genus level by Virgilio et al. (2015) based on molecular markers which confirmed the findings of Krosch et al. (2012). It was further supported by works by San Jose et al. (2018), Dupuis et al. (2018) and Zhang et al. (2022). Hancock and Drew (2018) consider *Zeugodacus* as a subgenus of *Bactrocera*. David et al. (2017) described *Bactrocerabrevipunctata* David and Hancock from Maharashtra which was later transferred to genus *Zeugodacus* by Doorenweerd et al. (2018). David and Ramani (2019) studied the postabdominal structures of 16 species of *Zeugodacus* from India and performed a morphology based phylogenetic analysis of tribe Dacini wherein *Bactrocera* and *Dacus* Fabricius were monophyletic and *Zeugodacus* was polyphyletic, which might be due to the reason that only Indian species were included in the phylogenetic analysis. In this paper, four new species of *Zeugodacus* are described with illustrations of postabdominal structures. Two species of dacines, Bactrocera (Parazeugodacus) abbreviata (Hardy) and Dacus (Mellesis) vijaysegarani Drew & Hancock are recorded for the first time from India. An illustrated key to 34 species of *Zeugodacus* from India is also included.

## ﻿Materials and methods

Specimens deposited in the following museums have been studied: Natural History Museum, London, United Kingdom (**NHM**) and National Insect Museum, ICAR- National Bureau of Agricultural Insect Resources, Bengaluru, India (**NIM**).

Images of specimens, epandrium, and ovipositor were taken using a Leica DFC 420 camera mounted on a Leica M205A stereo zoom microscope; images of glans of phallus, aculeus tip and spicules on eversible membrane were taken using an 8 MP camera temporarily attached to a Leica DM 1000 compound research microscope, Olympus DP 23 attached to BX51 and Olympus SC 50 attached to BX 43; the images were stacked and combined to a single image using Combine ZP (Hadley 2011). Measurements of male and female genitalia were taken using Leica Automontage Software, LAS 3.4. Terminology adopted here follows White et al. (1999) except for wing terminology which follows Cumming and Wood (2017).

One hind leg was removed from one specimen of *Z.momordicae* and used for DNA extraction. The DNA extraction was performed using a DNeasy Blood and Tissue Kit (Qiagen India Pvt. Ltd.) following the manufacturers’ instruction. For the molecular study, the standard DNA barcoding region of the mitochondrial COI gene was sequenced, and the PCR was performed using the Universal COI primers (LCO1490/HCO2198) (Hebert et al. 2003). The sequence was annotated using NCBI Blast tools and submitted to the NCBI GenBank Database where an accession number was obtained (*Z.momordicae*- OQ353070).

## ﻿Taxonomic account

### ﻿ Zeugodacus

Taxon classificationAnimaliaDipteraTephritidae

Hendel, 1927

B3DDDDE6-5DA6-52ED-B8F1-ABCA6E918585

Zeugodacus Hendel, 1927: 26. Raised to genus level by Virgilio et al. 2015: 177. Type species: Dacuscaudatus Fabricius, 1805: 276. Type locality: Indonesia, Java.

#### Diagnosis.

Abdominal tergites free, scutum with medial postsutural vitta except for few species in several subgenera including *Parasinodacus* Drew & Romig, *Paradacus* Perkins and some species of *Sinodacus* Zia, sternite 5 of male with shallow/flat posterior emargination. In males, epandrium distinctly bulb-shaped in posterior view, proctiger hyaline, triangular (when uninflated) smaller than epandrium, lateral surstylus longer than epandrium (profile view); posterior lobe of lateral surstylus 5–6× longer than anterior lobe. Phallus with well-developed acrophallus (single semi-tubular lobe) and patterned/granulated praeputium. Dorsal sclerite of glans without hexagonal pattern. Aculeus dorsoventrally flattened with four pairs of preapical setae (David and Ramani 2019). *Zeugodacus* is similar to *Bactrocera* and *Dacus* in general appearance as they are wasp mimics and are characterised by the presence of reddish-brown to black colour with yellow vittae and markings. It can be differentiated from *Dacus* by the presence of free abdominal tergites and by the presence of four pairs of preapical setae; from *Bactrocera* by the shallow/ flat emargination sternite 5 in males, posterior lobe of lateral surstylus 5–6× longer than anterior lobe and patterned acrophallus.

### ﻿Key to species of *Zeugodacus* Hendel from India

**Table:** 

1	Medial postsutural vitta present (Figs 10–20)	**6**
–	Medial postsutural vitta absent (Fig. 9)	**2**
2	Scutum black	**3**
–	Scutum reddish-brown, lateral postsutural vitta absent (Fig. 86), costal band broad overlapping vein R_2+3_, expanded into a broad apical spot (Fig. 89)	***Z* . *sinuvittatus* David & Abhishek, sp. nov.**
3	Lateral postsutural vitta absent, costal band overlapping vein R_2+3_ expanded slightly towards apex (Drew and Romig 2013: fig. 282)	***Z* . *binoyi* Drew**
–	Lateral postsutural vitta present, prescutellar setae present, costal band confluent with vein R_2+3_	**4**
4	Forefemur entirely black, 0.75 of mid and hind femur black (Fig. 43)	**5**
–	Forefemur fulvous with apical black spot, mid and hind femur fulvous with apical black spots (Fig. 21)	***Z* . *duplicatus* (Bezzi)**
5	Scutum with a yellow spot anterior to notopleural suture, prescutellar acrostichal seta absent	***Z* . *incisus* (Walker)**
–	Scutum without a yellow spot anterior to notopleural suture, prescutellar acrostichal seta present	***Z* . *momordicae* David & Ajaykumara, sp. nov.**
6	Postsutural supra-alar seta absent (Figs 13, 14)	**7**
–	Postsutural supra-alar seta present (Figs 15–20)	**9**
7	Costal band continuous, confluent with vein R_2+3_, not expanded into an apical spot (Fig. 36), prescutellar acrostichal seta present	**8**
–	Costal band discontinuous with a broad apical spot, prescutellar acrostichal seta absent (Drew and Romig 2013: fig. 9)	***Z* . *apicalis* (de Meijere)**
8	Face fulvous without any markings (Fig. 1), notopleuron yellow (Fig. 13)	***Z* . *trilineatus* (Hardy)**
–	Face with two separate black spots (Fig. 7), notopleuron black (Fig. 14)	***Z* . *scutellarius* (Bezzi)**
9	Prescutellar acrostichal seta absent (Fig. 12)	**10**
–	Prescutellar acrostichal seta present (Figs 15–20)	**12**
10	Abdomen oval shaped (Fig. 26); apical spot on wing does not cross vein M (Fig. 33)	***Z.havelockiae* Drew & Romig**
–	Abdomen club-shaped (Fig. 25); apical spot on wing crosses vein M	**11**
11	Yellow spot anterior to transverse suture as broad as notopleuron, lateral postsutural vitta absent, if present, not extending beyond postsutural supra-alar seta, apical spot on wing not reaching apices of vein R_2+3_ and dm-cu crossvein basally (Drew and Romig 2013: fig. 261)	***Z* . *hochii* (Zia)**
–	Yellow spot anterior to transverse suture narrower than notopleuron, lateral postsutural vitta prominent and narrows to end before postalar seta (Fig. 12), apical spot broad and reaching apices of vein R_2+3_ and dm-cu (Fig. 29)	***Z* . *brevipunctatus* (David & Hancock)**
12	Costal band narrow, confluent with vein R_2+3,_ either continuous or discontinuous, not expanded apically (Figs 31, 35, 36)	**13**
–	Costal band broad, overlapping vein R_2+3_, usually expanded into broad apical spot (Figs 30, 33, 34, 37, 38)	**26**
13	Costal band discontinuous (Fig. 31)	**14**
–	Costal band continuous	**15**
14	Scutellum shining black except for small yellow anterolateral corners (Drew and Romig 2013: fig. 281)	***Z.biguttatus* (Bezzi)**
–	Scutellum fully yellow without any black markings	***Z.freidbergi* White**
15	Scutum predominantly black with narrow lateral and medial postsutural vittae (Figs 15, 20)	**16**
–	Scutum black or brown with broad lateral and medial postsutural vittae (Figs 16–19)	**22**
16	Scutellum yellow with apical black spot (Fig. 15) or broad black band dividing it into two (Fig. 20)	**17**
–	Scutellum predominantly yellow without apical black spot or markings except for a narrow black basal band (Fig. 10)	**20**
17	Scutellum with broad black band dividing it into two yellow spots (Fig. 20)	***Z.assamensis* (White)**
–	Scutellum with an apical black spot (Fig. 15)	**18**
18	Face with two separate black triangular spots (Fig. 8)	***Z.scutellaris* (Bezzi)**
–	Face either black or with broad black transverse bands connecting spots (Figs 40, 41)	**19**
19	Medium sized flies (5.4–5.7 mm), face entirely black in males, female with distal half black, abdominal tergites 3–5 fully black	***Z.umiam* David & Kennedy, sp. nov.**
–	Large sized flies (7–8 mm), face with a transverse band connecting spots, abdominal tergites 3–5 with black markings restricted to lateral regions (Drew and Romig 2013: fig. 309)	***Z.hoabinhiae* Drew & Romig**
20	Only apical scutellar setae present	**21**
–	Apical and basal scutellar setae present	***Z.atrifacies* (Perkins)**
21	Face entirely black (Fig. 6), forefemur wholly black	***Z.diaphorus* (Hendel)**
–	Face with black spots, forefemur fuscous, not black (Drew and Romig 2013: fig. 370)	***Z.yoshimotoi* (Hardy)**
22	Anepisternal stripe broad touching postpronotal lobe, katepisternum with a broad yellow transverse marking, anepisternal stripe inverted L-shaped (Fig. 22)	***Z* . *gavisus* (Munro)**
–	Anepisternal stripe not touching postpronotal lobe, katepisternum with narrow yellow spot, anepisternal stripe triangular (Figs 23, 24, 79)	**23**
23	Abdomen reddish brown without any medial longitudinal band (Fig. 26), face with transverse marking interrupted medially (Fig. 5)	***Z* . *semongokensis* Drew & Romig**
–	Abdomen yellow with black transverse and longitudinal markings (Figs 27, 28, 80)	**24**
24	Face fulvous with two separate black spots (Fig. 77), medial postsutural vitta broadened posteriorly (nose-shaped) (Fig. 78), abdomen with medial black longitudinal band interrupted (Fig. 80)	***Z.nasivittatus* David & Abhishek, sp. nov.**
–	Face either fulvous or with transverse band (Figs 3, 4), medial postsutural vitta broad or narrow (Figs 17, 18) but not nose shaped, abdomen usually with medial vitta continuous (Figs 27, 28)	**25**
25	Medial postsutural vitta broadened basally and narrowed apically (Fig. 17), pecten of cilia present in male (Fig. 27), face with a black transverse band in both sexes (Fig. 4)	***Z.caudatus* (Fabricius)**
–	Medial postsutural vitta narrowed at both ends (Fig. 18), pecten of cilia absent in male (Fig. 28), face fulvous in male (Fig. 2), with a transverse band in female (Fig. 3)	***Z* . *diversus* (Coquillett)**
26	Costal band broad, confluent with R_4+5_ with or without apical expansion (Figs 30, 32, 34)	**27**
–	Costal band narrow, not confluent with R_4+5_; overlapping vein R_2+3_ or confluent with R_2+3_ (Figs 37, 38)	**30**
27	Wing with prominent subapical band and radial medial band (Fig. 30)	***Z.cucurbitae* (Coquillett)**
–	Wing without subapical band and radial-medial band (Figs 32, 34)	**28**
28	Scutellum with apical black spot (Figs 11, 16, 19)	**29**
–	Scutellum without apical black spot, costal band broadly confluent with R_4+5_, not expanded into an apical spot (Drew and Romig 2013: fig. 367)	***Z.vultus* (Hardy)**
29	Postpronotal lobe fuscous (Fig. 11), all femora with fuscous markings (Fig. 24), wing with costal band expanded to broad apical spot (Fig. 34)	***Z.watersi* (Hardy)**
–	Postpronotal lobe fulvous (Fig. 16), femora yellow (Fig. 23), wing with dark fuscous markings in apical region connected to anal streak (Fig. 32)	***Z.fuscoalatus* (Drew & Romig)**
30	Scutellum with apical black spot (Fig. 19)	***Z.signatus* (Hering)**
–	Scutellum without apical black spot	**31**
31	Costal band confluent with vein R_2+3_ (Fig. 37)	**32**
–	Costal band overlapping vein R_2+3_ (Fig. 38)	**33**
32	Lateral postsutural vitta parallel sided, not tapering posteriorly	***Z.zahadi* (Mahmood)**
–	Lateral postsutural vitta narrowing posteriorly (Drew and Romig 2013: fig. 363)	***Z.trivandrumensis* (Drew & Romig)**
33	All femora fulvous, anepisternal stripe broad reaching notopleural seta dorsally, supernumerary lobe weak in males (Drew and Romig 2013: fig. 282)	***Z.bogorensis* (Drew & Romig)**
–	All femora with preapical spots, wing in male with well-developed supernumerary lobe.	***Z.tau* (Walker)**

**Figures 1–8.**
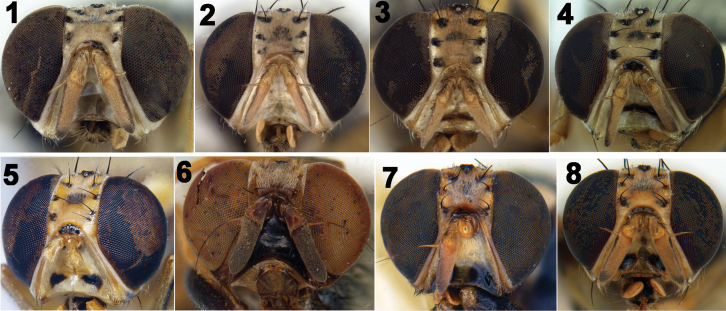
Heads of Tephritidae**1***Z.trilineatus* (Hardy) **2** male of *Z.diversus* (Coquillett) **3** female of *Z.diversus* (Coquillett) **4***Z.caudatus* (Fabricius) **5** Z. *semongokensis* (Drew & Romig) **6***Z.diaphorus* (Hendel) **7***Z.scutellarius* (Bezzi) **8***Z.scutellaris* (Bezzi).

**Figures 9–20.**
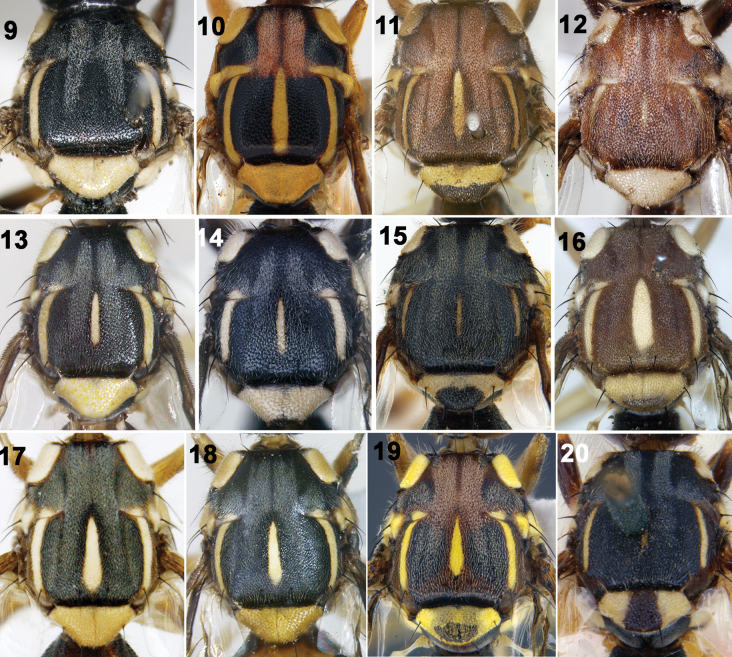
Thorax (dorsal view) of Tephritidae**9***Z.incisus* (Walker) **10***Z.gavisus* (Munro) **11***Z.watersi* (Hardy) **12***Z.brevipunctatus* (David & Hancock) **13***Z.trilineatus* (Hardy) **14***Z.scutellarius* (Bezzi) **15***Z.scutellaris* (Bezzi) **16***Z.fuscoalatus* (Drew & Romig) **17***Z.caudatus* (Fabricius) **18***Z.diversus* (Coquillett) **19***Z.signatus* (Hering) **20***Z.assamensis* (White).

**Figures 21–28.**
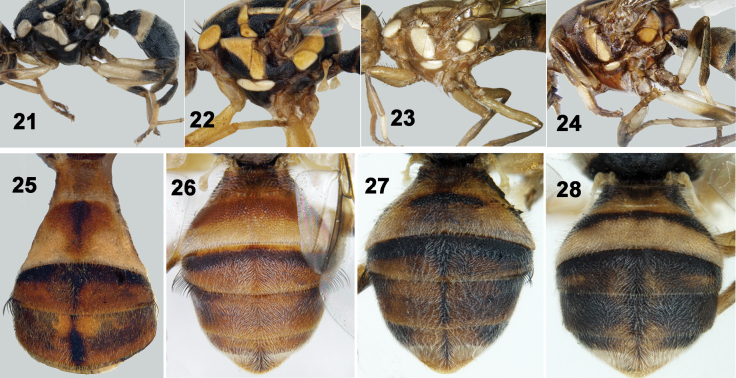
**21–24** Thorax (lateral view) and legs **25–28** Abdomen (dorsal view) of Tephritidae**21***Z.duplicatus***22***Z.gavisus***23** Z. *fuscoalatus***24***Z.watersi***25***Z.brevipunctatus***26***Z.semongokensis***27***Z.caudatus***28***Z.diversus*.

**Figures 29–38.**
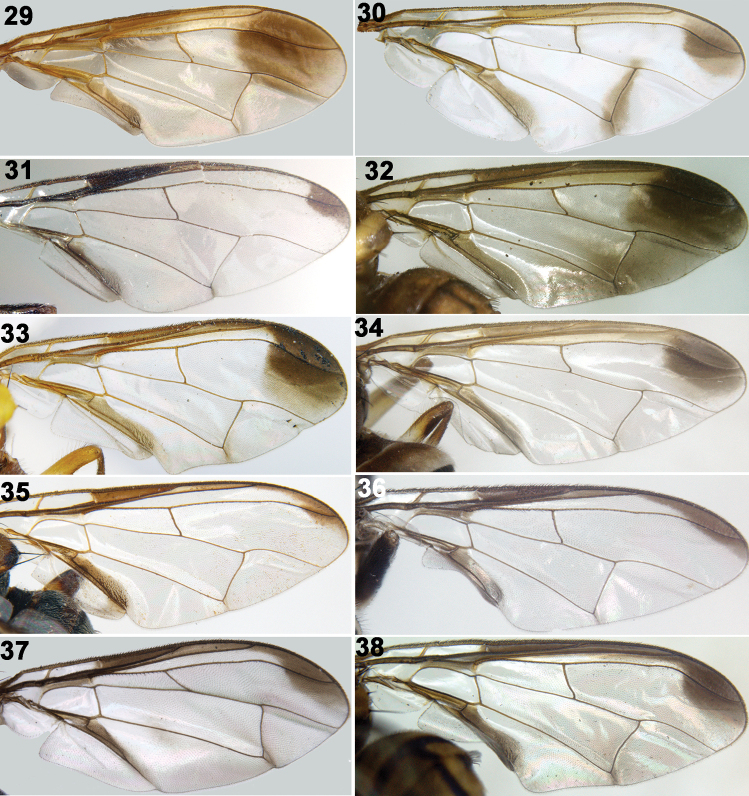
Wings of Tephritidae**29***Z.brevipunctatus***30***Z.cucurbitae***31***Z.freidbergi***32***Z.fuscoalatus***33***Z.havelockiae***34***Z.watersi***35***Z.scutellaris***36***Z.scutellarius***37***Z.zahadi***38***Z.tau*.

### ﻿New species descriptions

### ﻿ Zeugodacus (Parasinodacus) momordicae

Taxon classificationAnimaliaDipteraTephritidae

David & Ajaykumara sp. nov.

0DBC5A96-4B39-5B83-8C7D-8861DFD6567F

https://zoobank.org/D8024CC3-CE48-4C83-B2DC-D737D87FD34F

Figs 39 , 40–49 , 50–57 , 58–60

#### Type locality.

India: Arunachal Pradesh, Upper Siang, Padu.

#### Type material.

***Holotype*** female, pinned. Original label: “INDIA: Arunachal Pradesh, Upper Siang, Padu, 29. ix. 2022, David, K. J.” ***Paratypes*.** 20♀♀, 3♂♂, India: Arunachal Pradesh, Upper Siang, Padu, 15. ix. 2022, Ajaykumara, K. M.; 15♀♀, 3♂♂, 1 larva, India: Arunachal Pradesh, Upper Siang, Padu, 29. ix. 2022, David, K. J. (deposited at NIM).

#### Other material examined.

1♀, Formosa, Kagi, 19.08.07, H. Sauter, S.(first label), *Chaetodacuscilifer* Hend.♀ det. M. Hering 1935 (second label) (NHM). 2♀♀, India, Meghalaya, Umiam, 19. v. 2017, Arensungla Pongen (NIM).

#### Diagnosis.

*Zeugodacusmomordicae* resembles *Z.incisus* in possessing black scutum, two transverse bands on face, continuous costal band and extensive femoral markings, but can be differentiated by the absence of yellow spot anterior to lateral vittae along transverse suture and presence of prescutellar acrostichal setae. It can be differentiated from *Bactroceraablepharus* (Bezzi) by the presence of prescutellar acrostichal setae and face with two transverse bands. It can be differentiated from *Z.cilifer* (Figs 61–65, 69) by the aculeus shape and spicules on distal end of eversible membrane as discussed below. Aculeus tip is elongate, parallel sided and not tapering abruptly beyond the preapical conical flange (width of the conical projection- 0.06 mm) and length of aculeus after the preapical flange is 0.21 mm in *Z.cilifer* (Figs 66, 67), whereas in *Z.momordicae*, aculeus is tapering abruptly beyond the preapical conical flange (width of the conical projection -0.08 mm) (Figs 56, 57) and length of aculeus after the preapical flange is 0.15–0.18 mm. Spicules on *Z.cilifer* are conical with single projection with a shorter base (Fig. 68), whereas *Z.momordicae* (Fig. 54) possess broader conical spicules.

#### Description.

**Female.** Medium sized species (wing length 4.37–5.45 mm), face with two broad black bands. Scutum black with yellow lateral postsutural vitta ending beyond intra-alar seta, anepisternal stripe broad reaching anterior notopleural seta dorsally, continued as a small transverse marking on katepisternum. Wing hyaline with costal band continuous from cell sc to the apex of the wing and confluent with vein R_2+3_, anal streak well developed. Abdomen predominantly black with a narrow transverse fulvous band on tergites 1 and 2 (in few specimens all tergites black). Females with two spermatheca, aculeus pointed with preapical projection.

***Head*.** Frons fulvous with fuscous markings on anteriomedial hump and around bases of frontal and orbital setae, all setae black; 2 pairs of frontal setae and 1 pair of orbital setae, lunule black. Ocellar triangle and vertex black, ocellar setae vestigial. Face (Figs 40, 41) fulvous with two broad transverse bands (elongate spots in antennal furrow connected by broad transverse band and a broad black band below the antennal sockets). Scape, pedicel fulvous, first flagellomere dark fuscous on outer side and apex, arista non plumose, combined length of pedicel and flagellomere is slightly longer than vertical length of face. Gena fulvous with prominent black patch and a seta. Occiput black, fulvous along eye margins; lateral and medial vertical seta present, occipital row without prominent black postocular setae**. *Thorax*** (Figs 42, 43). wholly black with yellow lateral postsutural vittae extending beyond intra-alar seta, medial vitta lacking; pleura black. Yellow marking as follows; postpronotal lobe, notopleuron, anepisternal stripe (reaching anterior notopleural seta dorsally) continued to katepisternum as a transverse spot; anatergite (posterior apex black); anterior 0.60 of katatergite (remainder black). Scutellum yellow with two scutellar setae. Chaetotaxy: scutellar seta, 1; prescutellar acrostichal seta, 1; intra-alar seta, 1; postsutural supra-alar seta, 1; postalar seta, 1; anepisternal seta, 1; anterior notopleural seta, 1; posterior notopleural seta, 1; scapular setae, 2. Coxa and trochanter black; whole fore femur; 0.75 of mid femur; 0.25 of hind femur black; remainder black. Fore and mid tibiae fulvous/yellow, hind tibia black, all tarsal segments fulvous (Fig. 43). ***Wing*** (4.37–5.45 mm) predominantly hyaline, cells bc and c hyaline, cell sc dark fuscous, costal band confluent with vein R_2+3_, slightly expanded apically, anal streak as broad as cell cua_1_ extending till apex of its extension, supernumerary lobe developed (Figs 48, 49). ***Abdomen*** (Figs 44–47). Abdominal segments entirely black except for a narrow fulvous band on tergite 2 apically (in few specimens all tergites black).

**Male.** Similar to female except for face (Fig. 40) which is nearly black in few males with a narrow longitudinal fulvous line separating the bands, costal band discontinuous in few male specimens examined, sternite 5 in males black with shallow concavity, pecten present on tergite 3.

***Female genitalia*.** Oviscape conical (Fig. 53), dorsoventrally flattened, basal half dark fuscous, apical half fulvous; eversible membrane twice as long as oviscape (1.69 mm), spicules on distal end of eversible membrane (2.98 mm) with medial conical projection with a wider base (Fig. 54); aculeus (1.36 mm) shorter than eversible membrane with conical preapical flange, needle-shaped aculeus tip (Figs 56, 57); spermatheca dark brown, tightly coiled (Fig. 55).

**Figure 39.**
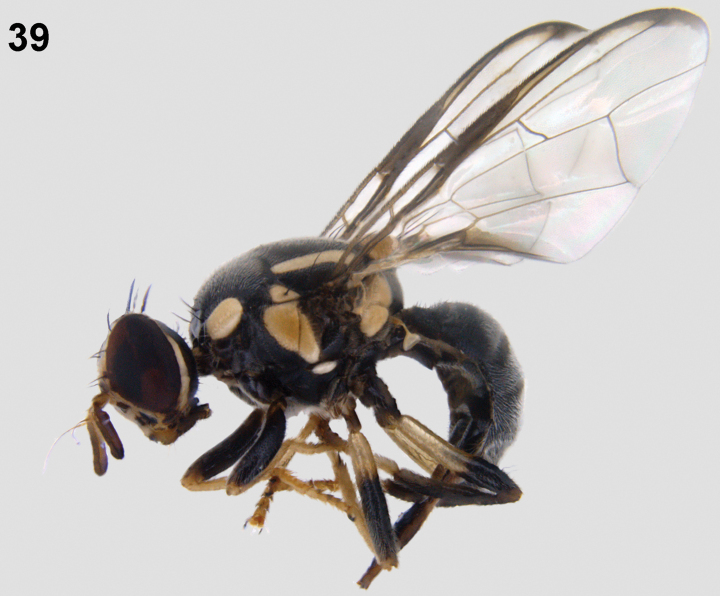
Habitus (lateral) of female *Zeugodacusmomordicae* David & Ajaykumara, sp. nov.

**Figures 40–49.**
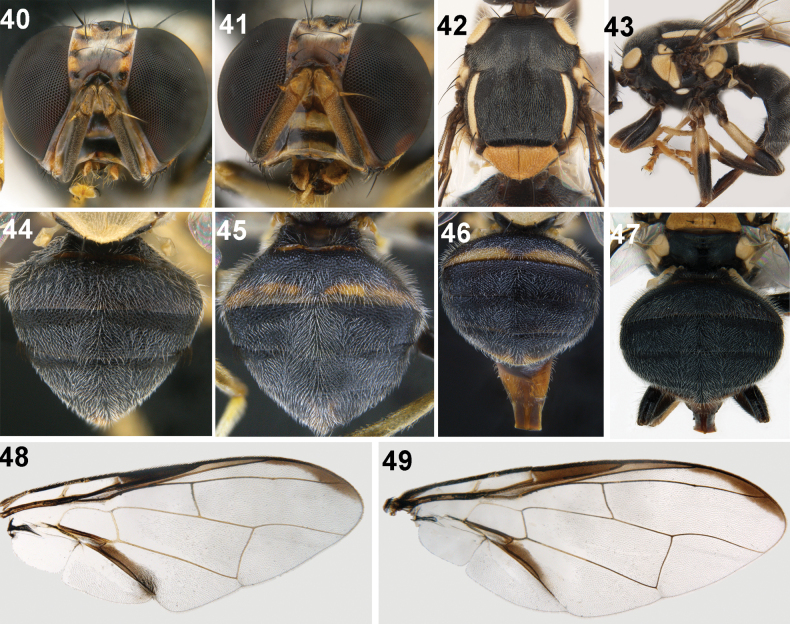
*Zeugodacusmomordicae* David & Ajaykumara, sp. nov. **40** head frontal view (male) **41** head frontal view (female) **42** scutum **43** thorax (lateral view) and legs **44, 45** male abdomen (dorsal view) **46, 47** female abdomen (dorsal view) **48** wing (male) **49** wing (female).

**Figures 50–57.**
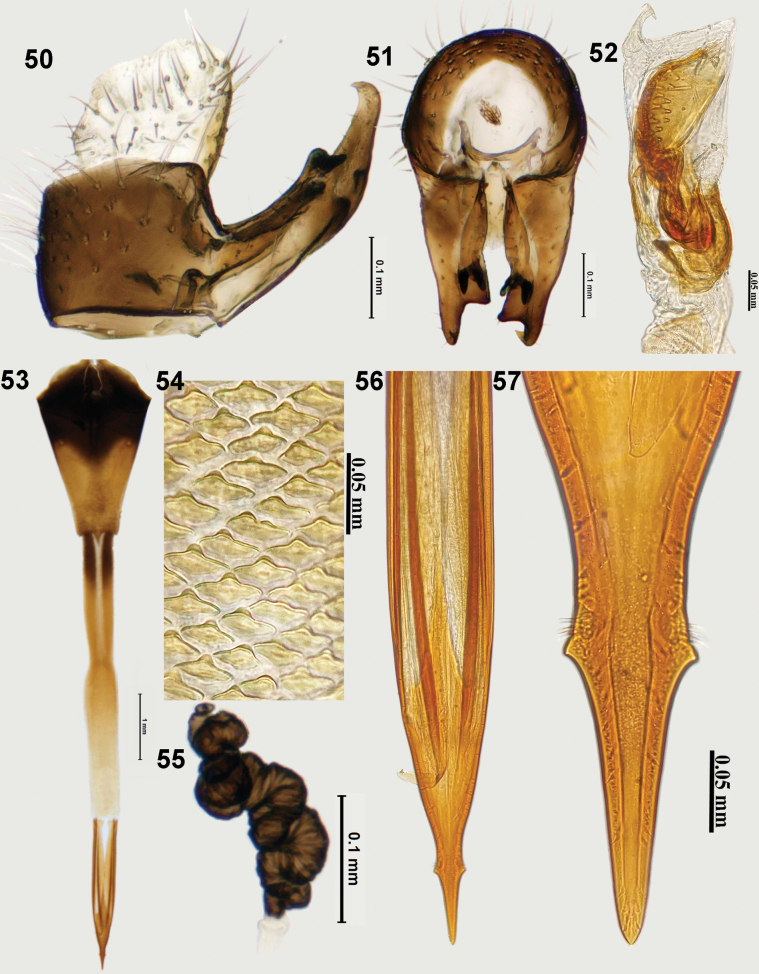
Postabdominal structures of *Zeugodacusmomordicae* David & Ajaykumara, sp. nov. **50** epandrium and surstyli (lateral view) **51** epandrium and surstyli (posterior view) **52** glans of phallus **53** ovipositor **54** spicules on distal end of eversible membrane **55** spermatheca **56** aculeus **57** aculeus tip.

***Male genitalia*.** Epandrium quadrate (profile view), lateral surstylus longer than epandrium; posterior lobe of surstylus 10× longer than anterior lobe (Fig. 50); proctiger hyaline as broad as but, shorter than epandrium; medial surstylus shorter than lateral surstylus with well-developed pair of equal sized prensisetae (Fig. 51). Phallus 3.15 mm long, glans of phallus well sclerotised with 2–3 rows of spine like projections on acrophallus dorsally (Fig. 52), subapical lobe T-shaped, preglans lobe present.

**Figures 58–60.**
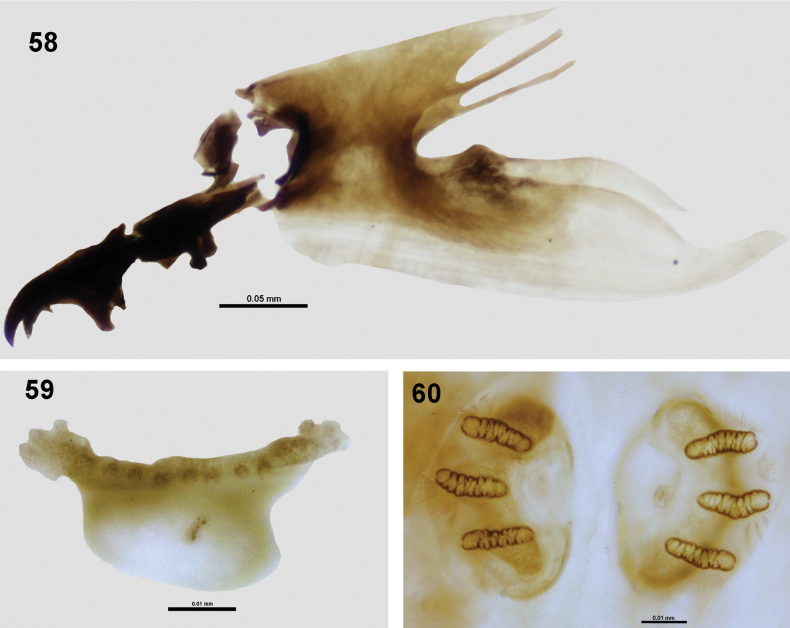
Larval morphology of *Zeugodacusmomordicae* David & Ajaykumara, sp. nov. **58** cephalopharyngeal skeleton **59** anterior spiracle **60** posterior spiracles.

***III instar larva*.** Creamy white, tapered anteriorly, blunt posteriorly. Cephalopharyngeal skeleton (Fig. 58): Mandible pointed, with short preapical tooth smaller than apical tooth, ventral apodeme prominent, mandibular neck well developed; hypopharyngeal sclerite shorter than mandible narrowing distally, with well-developed hypopharyngeal bridge in the centre, parastomal bar well developed covering the entire length of hypopharyngeal sclerite, labial sclerite present, anterior sclerite well developed (detached while dissecting); pharyngeal sclerite with well-developed dorsal and ventral cornua, ventral bridge lacking. Anterior spiracle with 17 tubules (Fig. 59); slits of posterior spiracle arranged parallel to each other with well-developed dorsal, lateral and ventral spiracular bundles (Fig. 60).

#### Etymology.

The species name is derived from the genus name *Momordica* in the genitive case.

#### Host plant.

Flies were collected on spiny/spine gourd (Figs 70–72); female flies were observed ovipositing inside unopened male flower buds of spiny gourd (Figs 73, 74), *Momordicadioica* Roxb ex Wild. Infested flower buds were having a withered appearance with two or three larvae inside (Figs 75, 76). They were reared up to the adult stage to confirm it as the host.

#### DNA Barcode.

NCBI Accession number OQ353070 (1♀, INDIA: Arunachal Pradesh, Upper Siang, Padu, 29. ix. 2022, David, K.J.). The partial gene sequence of mt-COI of Indian specimen was subjected to similarity search (BLAST-N) in NCBI database which revealed 99% similarity with *Zeugodacuscilifer* reported from Thailand and China, however 97.87% similarity was observed with *Z.cilifer* from Taiwan.

#### Remarks.

**Figures 61–65.**
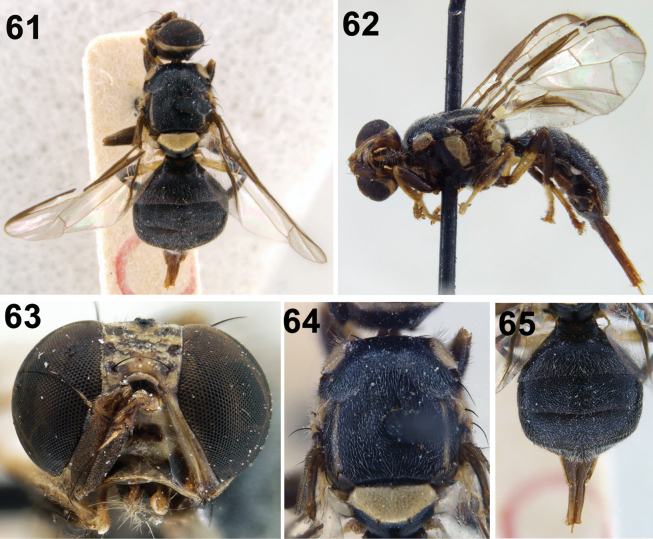
Zeugodacus (Parasinodacus) cilifer (Hendel) **61** habitus (dorsal) **62** habitus (lateral) **63** head (frontal view) **64** scutum (dorsal view) **65** abdomen.

Nair et al. (2018; 2021) and Pongen et al. (2023) reported *Zeugodacuscilifer* from Tripura and Meghalaya as a pest of flowers of spiny gourd, respectively. Examination of the postabdominal structure of female of *Z.cilifer* collected from Taiwan (Figs 61–65), the type locality in 1907, deposited at Natural History Museum, London and specimens collected from Pasighat, Arunachal Pradesh and Meghalaya, India revealed that specimens from India are different in the morphology of aculeus and spicules on distal end of eversible membrane as mentioned in the diagnosis, hence it is here described as a new species. Hence records of *Z.cilifer* by Nair et al. (2018; 2021) and Pongen et al. (2023) are treated as misidentifications of *Z.momordicae*. It is placed in *Zeugodacus* due to shallow/flat posterior emargination of sternite 5 in males, posterior lobe of lateral surstylus much longer than anterior lobe and patterned acrophallus. It is placed in subgenus Parasinodacus as it possesses only two scutellar setae and scutum is devoid of medial postsutural vitta.

**Figures 66–69.**
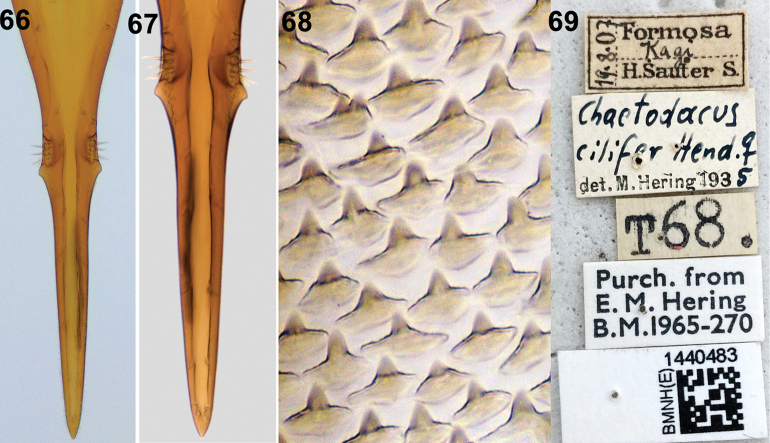
Zeugodacus (Parasinodacus) cilifer (Hendel) **66, 67** aculeus tip **68** spicules on distal end of eversible membrane **69** label data.

**Figures 70–76.**
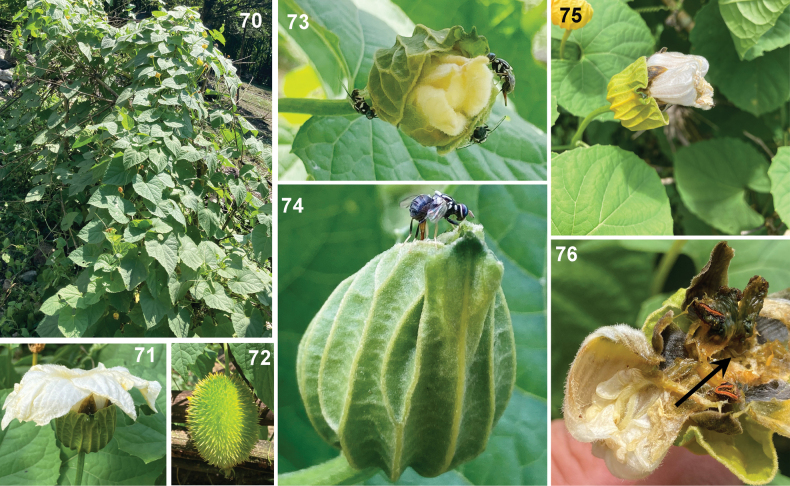
Field infestation of Zeugodacus (Parasinodacus) momordicae David & Ajaykumara, sp. nov. **70** habitus of host plant, Spiny gourd, *Momordicadioica***71** healthy male flower **72** fruit **73** males and females of *Z.momordicae* on male flower bud of spiny gourd **74** female fly of *Z.momordicae* ovipositing into male flower buds **75** infested male flower **76** cut opened infested flower with maggots.

### ﻿ Zeugodacus (Zeugodacus) nasivittatus

Taxon classificationAnimaliaDipteraTephritidae

David & Abhishek sp. nov.

E7CB4899-B03D-5744-A2DB-F036D244A97C

https://zoobank.org/BA85A422-419F-485A-BCBC-AC73D7BB27C6

Figs 77–84

#### Type locality.

India, Meghalaya, Umiam.

#### Type material.

***Holotype*** male, pinned. Original label: “INDIA, Meghalaya, Umiam, 11.vii.2023, Kennedy N.” ***Paratype*** 1♂, India: Meghalaya, Umiam, 11.vii.2023, Kennedy N., attracted to cue lure (deposited at NIM).

#### Diagnosis.

It is similar to *Zeugodacushengsawadae* Drew & Romig and *Z.tebeduiae* Drew & Romig in possessing broad medial postsutural vitta and costal band confluent with vein R_2+3_, but can be easily separated from *Z.hengsawadae* by the entirely fulvous femora without any preapical spots, absence of basal scutellar seta and shape of the medial vitta; from *Z.tebeduiae* by its smaller size (wing length 4.5 mm), absence of elongate narrow facial spots and basal scutellar setae. It can be differentiated from *Z.flavoverticalis* Drew & Romig by the absence of broad transverse marking on katepisternum, presence of slightly expanded costal band towards apex and yellow abdominal tergites with narrow medial and longitudinal bands.

**Figures 77–84.**
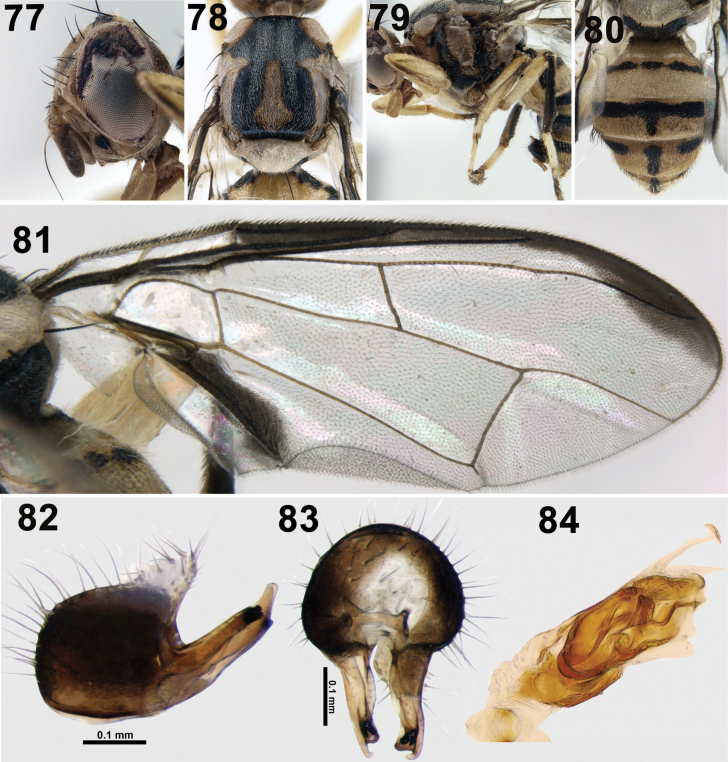
*Zeugodacusnasivittatus* David & Abhishek, sp. nov. **77** head (lateral) **78** scutum (dorsal view) **79** thorax (lateral) **80** abdomen **81** wing **82** epandrium (lateral) **83** epandrium (posterior) **84** glans of phallus.

#### Description.

**Male.** Medium sized species (5.7–5.8 mm); face fulvous with two separate black spots; scutum black colour with a broad lateral postsutural yellow vitta (0.16–0.18 mm wide) ending behind intra-alar seta; notopleuron and postpronotal lobe yellow, prominent yellow spot anterior to notopleural suture; anepisternal stripe reaching anterior notopleural seta dorsally; scutellum without black basal band; wing predominantly hyaline with narrow costal band confluent with R_2+3_, anal streak wide, dense aggregation of microtrichia around A_1_+Cu_2_; abdominal tergites 3–5, orange-brown with a narrow longitudinal black discontinuous band (0.17 mm), lateral regions of tergites 3–5 with small, fuscous markings.

***Head*** (Fig. 77): Height 1.21 mm. Frons length 1.67× breadth; fulvous with fuscous marking on anteriomedial hump and around bases of frontal and orbital setae, all setae black: three pairs of frontal setae and one pair of orbital setae; lunule fulvous. Ocellar triangle and vertex black. Face fulvous with two separate black spots (0.16 mm long) on antennal furrows. Scape (0.12 mm long) and pedicel (0.21 mm long) fulvous, first flagellomere (0.51 mm long) dark fuscous on outer side and apex, arista non plumose, combined length of pedicel and flagellomere as long as the vertical length of face. Gena fulvous without a black marking, genal seta present. Occiput light fuscous, fulvous along eye margins; lateral and medial vertical setae present, occipital row without stout black setae. ***Thorax*** (Figs 78, 79). scutum black (1.75 mm long, 1.74 mm wide) without lanceolate markings. Pleura black in ground colour with red-brown markings anterior to anepisternal stripe, katepisternum and anepimeron. Yellow markings as follows: postpronotal lobe, notopleuron, anepisternal stripe reaching anterior notopleural seta dorsally and continuing to katepisternum as a transverse spot; anatergite (posterior apex black); anterior 3/4 of katatergite (remainder black); broad parallel-sided lateral postsutural vitta ending after intra-alar seta. Medial longitudinal postsutural yellow vitta present (nose shaped). Scutellum yellow without narrow black basal band. Chaetotaxy: scutellar seta, 1; prescutellar acrostichal seta, 1; intra-alar seta, 1; postsutural supra-alar seta, 1; postalar seta, 1; anepisternal seta, 1; anterior notopleural seta, 1; posterior notopleural seta, 1; scapular setae, 2. Coxa fulvous, trochanter light fulvous; all femora fulvous without apical black markings; fore femur without small oval spot, apex of mid femur with faint infuscation; hind femur with prominent black apex. Fore and mid tibiae light fuscous at base, hind tibia dark fuscous, all tarsal segments fulvous. ***Wing*** (Fig. 81). Length, 4.65 mm, cells bc and c hyaline; microtrichia in outer corner of cell c only; remainder of wing hyaline except dark fuscous cell sc, costal band broad, confluent with R_2+3_ expanded slightly towards apex, extension of cell cua longer than cell cua, base of cell br with microtrichia, anal streak wide covering cell cua, with dense aggregation of microtrichia around A_1_+Cu_2_; supernumerary lobe well developed. ***Abdomen*** (Fig. 80). 2.81 mm long, 1.66 mm wide, oval, tergites free, tergites 1 and 2 fulvous, tergite 2 with a medial black spot. Tergite 3 reddish brown with a narrow, basal transverse black band. Tergites 3–5 with a narrow, discontinuous medial longitudinal black band and narrow, black lateral markings. Tergite 5 with inconspicuous ceromata, sternite 5 black with shallow concavity and pecten present on tergite 3.

***Male genitalia*.** Epandrium quadrate (profile view), lateral surstylus as long as epandrium; posterior lobe of surstylus 6–7× longer than anterior lobe (Fig. 82); proctiger hyaline, shorter than epandrium; medial surstylus shorter than lateral surstylus with well-developed pair of equal sized prensisetae (Fig. 83). Phallus short, 1.20 mm excluding glans of phallus (0.27 mm), glans of phallus well sclerotised with spine like projections on acrophallus (Fig. 84), subapical lobe T-shaped, preglans lobe present.

#### Etymology.

The species name is derived from Latin words *nasi vitta* which means nose-shaped vitta.

#### Host plant.

Not known.

#### Male parapheromone.

Cue lure.

#### Remarks.

This species is placed in *Zeugodacus* due to the shallow/flat posterior emargination of sternite 5 in males, posterior lobe of lateral surstylus much longer than anterior lobe and patterned acrophallus. It is placed in subgenus Zeugodacus as it possesses medial postsutural vitta, postsutural supra-alar, and prescutellar acrostichal seta.

### ﻿ Zeugodacus (Sinodacus) sinuvittatus

Taxon classificationAnimaliaDipteraTephritidae

David & Abhishek sp. nov.

96976936-2620-5643-B53E-CF4BD8CB1DD4

https://zoobank.org/424F5A47-0662-4551-A793-238FA7FBF63F

Figs 85–92

#### Type locality.

India, Himachal Pradesh, Totu, IARI substation, Totu.

#### Type material.

***Holotype*** male, pinned. Original label: “INDIA, Himachal Pradesh, Totu, IARI substation, Totu, 18.viii.2019, David, K. J.” (deposited at NIM).

#### Diagnosis.

*Zeugodacussinuvittatus* is similar to *Z.hochii* (Zia), *Z.infestus* (Enderlein) and *Z.brevipunctatus* David & Hancock in possessing reddish brown scutum, club shaped abdomen and wing with broad apical black spot. It can be differentiated from *Z.hochii* by the absence of medial postsutural vitta, face with separate black spots unlike transverse band, presence of discontinuous costal band slightly overlapping vein R_2+3_; from *Z.infestus* and *Z.brevipunctatus* by the absence of lateral and medial postsutural vitta, absence of postsutural supra-alar seta, narrow costal band interrupted in cell r_1_ and by the broad apical spot. Unlike *Z.brevipunctatus*, acrophallus of *Z.sinuvittatus* is fully patterned.

#### Description.

**Male.** Large sized species (wing length 7.05 mm); face fulvous with two elongate black markings in the antennal furrow and a medial longitudinal line; scutum reddish brown in ground colour without lateral and medial vitta, with broad quadrate black patches in presutural and postsutural areas, notopleuron and postpronotal lobe yellow, inconspicuous yellow spot anterior to notopleural suture; anepisternal stripe reaching midway between anterior notopleural seta and notopleuron; scutellum yellow; wing predominantly hyaline with costal band slightly overlapping vein R_2+3_, discontinuous towards apex of cell r_1_, with a broad apical spot covering the apex of cell r_2+3_, r_4+5_ and upper one-fourth of cell m, anal streak narrow, no dense aggregation of microtrichia around A_1_+Cu_2_; abdomen club shaped, tergite 2 with a prominent black semicircular spot, tergites 3–5 with dark fuscous lateral markings and a narrow medial longitudinal band.

**Figures 85–92.**
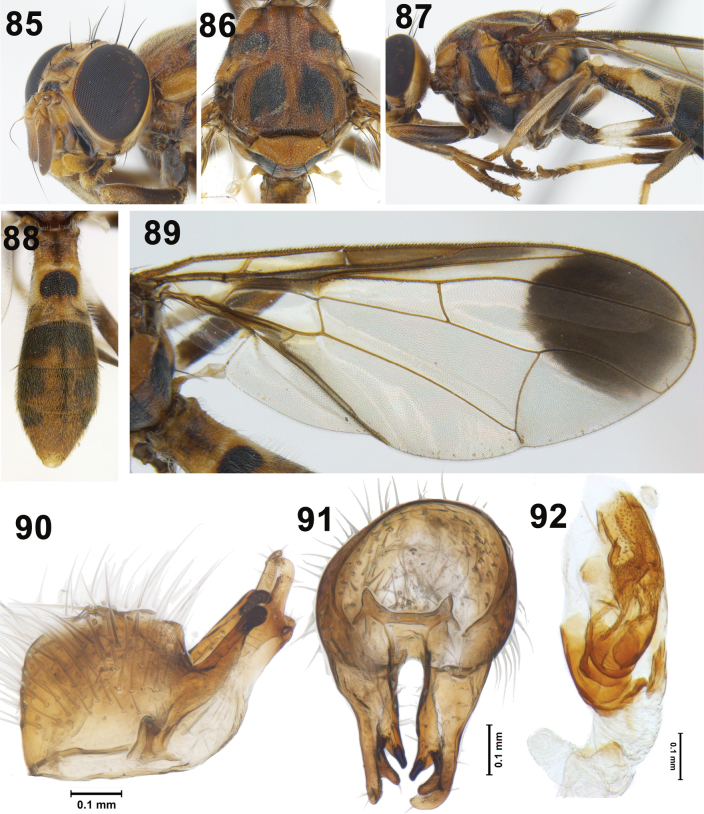
Zeugodacus (Sinodacus) sinuvittatus David & Abhishek, sp. nov. **85** head (lateral) **86** scutum (dorsal view) **87** thorax (lateral) **88** abdomen **89** wing **90** epandrium (lateral) **91** epandrium (posteriror) **92** glans of phallus.

***Head*** (Fig. 85). Height 1.60 mm. Frons length 1.85× breadth; fulvous with fuscous marking on anteriomedial hump and around bases of frontal and orbital setae, all setae black: two pairs of frontal setae and one pair of orbital setae; lunule black. Ocellar triangle black, vertex yellow. Face fulvous with two separate elongate black markings in antennal furrows and a medial longitudinal black line. Scape (0.23 mm long) and pedicel (0.22 mm long) fulvous, first flagellomere (0.74 mm long) dark fuscous on outer side and apex, arista non plumose, combined length of pedicel and flagellomere longer than the vertical length of face. Gena fulvous with a black marking and a seta. Occiput fulvous; lateral and medial vertical setae present, occipital row with three or four stout black setae. ***Thorax*** (Figs 86, 87). 2.18 mm long, 2.03 mm wide; scutum red brown with two black quadrate markings one each in presutural and postsutural area. Pleura red-brown in ground colour with black markings anterior to anepisternal stripe, katepisternum and anepimeron. Yellow markings as follows: postpronotal lobe, notopleuron, anepisternal stripe reaching midway between notopleuron and anterior notopleural seta and continuing to katepisternum as a transverse spot; anatergite (posterior apex black); anterior 3/5 of katatergite (remainder black). Scutellum yellow without narrow black basal band, subscutellum red-brown with black lateral margins. Chaetotaxy: scutellar seta, 1; intra-alar seta, 1; postalar seta, 1; anepisternal seta, 1; anterior notopleural seta, 1; posterior notopleural seta, 1; scapular setae, 1. Leg (Fig. 87): Coxa, trochanter dark fuscous, all femora with extensive fuscous markings; fore femur wholly fuscous, 0.80 of mid femur and 0.60 of hind femur fuscous; fore and hind tibiae fuscous, mid tibia fulvous, tarsal segments slight fuscous. ***Wing*** (Fig. 89). Length, 7.05 mm, cells bc and c hyaline; microtrichia in outer corner of cell c only; remainder of wing hyaline except dark fuscous cell sc, costal band overlapping vein R_2+3_, interrupted towards apical one-fourth of cell r_1_ and with a broad apical spot covering apex of cell r_2+3_, r_4+5_ and anterior one fourth of cell m, extension of cell cua longer than cell cua, base of cell br with microtrichia, anal streak narrow, confined to cell cua, lacks dense aggregation of microtrichia around A_1_+Cu_2_; supernumerary lobe weak. ***Abdomen*** (Fig. 88). 3.69 mm long, 1.92 mm wide, club shaped, tergites free, tergites 1 fulvous, tergite 2 reddish brown with a black semicircular marking, tergite 3 with broad, black basal band and pecten, tergites 4 and 5 with dark lateral margins and a narrow medial longitudinal band. Tergite 5 without prominent shining spots (ceromata).

***Male genitalia*.** Sternite 5 brown with shallow emargination, epandrium quadrate with lateral surstylus as long as epandrium, proctiger membranous, as wide as epandrium, epandrium sclerotised (Figs 90, 91), as long as wide (height 0.3 mm; width 0.34 mm) ; surstyli as long as epandrium, oblique, 0.38 mm long; posterior lobe of surstylus 6.2× longer than anterior lobe, aedeagus 4.60 mm long with glans of phallus (Fig. 92) 0.625 mm long. Three-quarters of glans heavily sclerotised with well-developed fully patterned acrophallus, praeputium, and subapical lobe.

#### Etymology.

The species name is derived from Latin words *sine* (= without) and *vitta* (= band), as the species lacks lateral and medial poststurural vitta on scutum.

#### Host plant.

Not known, collected by sweep netting on grapevine

#### Male parapheromone.

Not known.

#### Remarks.

This species is placed in *Zeugodacus* due to shallow/flat posterior emargination of sternite 5 in males, posterior lobe of lateral surstylus much longer than anterior lobe and patterned acrophallus. It is placed in subgenus Sinodacus as it lacks prescutellar acrostichal seta, basal scutellar seta and due the club-shaped abdomen.

### ﻿ Zeugodacus (Zeugodacus) umiam

Taxon classificationAnimaliaDipteraTephritidae

David & Kennedy sp. nov.

45502749-D8C7-501C-A5B8-2202A5DDD480

https://zoobank.org/3EDCB89D-2BBD-451C-B648-72CAC36605E7

Figs 93–101 , 102–109

#### Type locality.

India: Meghalaya, Umiam.

#### Type material.

***Holotype*** female, pinned. Original label: “INDIA: Meghalaya, Umiam, 06.vii.2021. Kennedy N.” ***Paratype***,1♂, India, Meghalaya, Bhoirymbong, Umiam,10.v.2023, Kennedy N, attracted to cue lure (deposited at NIM).

#### Diagnosis.

*Zeugodacusumiam* is similar to *Z.nigrifacies* (Shiraki) in possessing black face, fore femur entirely black, scutellum with broad black basal band and an apical spot but can be differentiated by the absence of subapical band, band on crossvein r-m and two scutellar setae. It can be distinguished from *Z.menglanus* (Yu, Liu & Yang) by the facial markings (wholly black in male; dorsal half black in female), two scutellar setae and lack of apical expansion in costal band. It is similar to *Z.diaphorus* in possessing apical scutellar spot, two pairs of scutellar setae and black face, but can be separated by the presence of broad black basal band on scutellum, narrow anepisternal stripe not reaching anterior notopleural seta dorsally.

#### Description.

**Female.** Medium sized, black species (wing length 5.65 mm); face posterior half black; scutum black with narrow yellow lateral postsutural vitta and medial vitta, lateral vitta ending before postalar seta, notopleuron and postpronotal lobe yellow, small yellow spot anterior to notopleural suture, anepisternal stripe not reaching anterior notopleural seta dorsally, scutellum yellow with a broad black basal band, with an apical black spot; wing predominantly hyaline with costal band confluent with vein R_2+3_, expanded slightly towards apex of cell r_2+3_ and r_4+5_, anal streak prominent; abdomen oval, all tergites black except tergite 2 with a broad fulvous band posteriorly, narrow fulvous bands in tergites 3–5.

**Figures 93–101.**
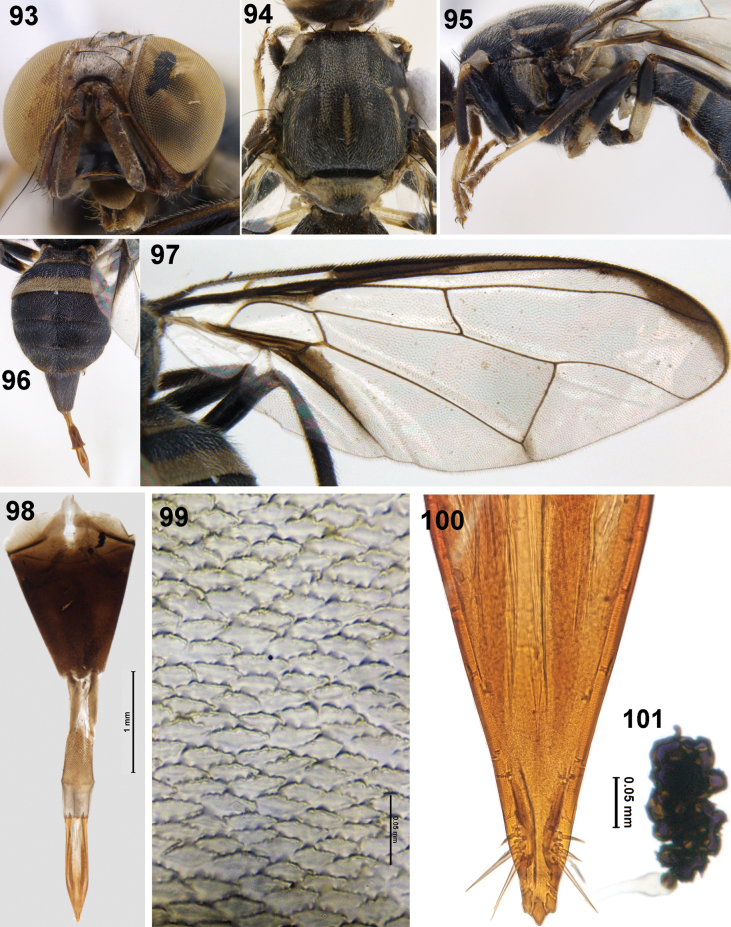
Female of *Zeugodacusumiam* David & Kennedy, sp. nov. **93** head (frontal view) **94** scutum (dorsal view) **95** thorax (lateral view) and legs **96** abdomen **97** wing **98** ovipositor **99** spicules on distal end of eversible membrane **100** aculeus **101** spermatheca.

***Head*** (Fig. 93): Height 1.32 mm. Frons length 1.2× breadth; fuscous, all setae black: two pairs of frontal setae and one pair of orbital setae; lunule black. Ocellar triangle, vertex black, face black in distal half, scape (0.12 mm long) and pedicel (0.14 mm long) fulvous, first flagellomere (0.58 mm long) dark fuscous on outer side and apex, arista non plumose, combined length of pedicel and flagellomere as long as the vertical length of face. Gena fulvous with a black marking and a seta, occiput black; lateral and medial vertical setae present. ***Thorax*** (Figs 94, 95): 2.18 mm long, 2.03 mm wide; scutum black with narrow yellow lateral postsutural vittae ending at postalar seta, medial vitta narrow. Yellow markings as follows: postpronotal lobe, notopleuron, anepisternal stripe reaching midway between notopleuron and anterior notopleural seta and continuing to katepisternum as a small transverse spot; anatergite (posterior apex black); anterior 3/5 of katatergite (remainder black). Scutellum yellow with a black basal band and an apical black spot, subscutellum black. Chaetotaxy: scutellar seta, 1; intra-alar seta, 1; postalar seta, 1; anepisternal seta, 1; anterior notopleural seta, 1; postsutural supra-alar seta,1; posterior notopleural seta, 1; scapular setae, 1. Leg (Fig. 95): Coxa, trochanter black, all femora with extensive fuscous markings; fore femur wholly black, 0.75 of mid femur and 0.50 of hind femur black; fore, mid and hind tibiae black, tarsal segments fulvous. ***Wing*** (Fig. 97): Length, 5.65 mm, cells bc and c hyaline; microtrichia in outer corner of cell c only; remainder of wing hyaline except dark fuscous cell sc, dark fuscous narrow costal band confluent with vein R_2+3_ expanded slightly towards apex, extension of cell cua as long as cell cua, base of cell br with microtrichia, anal streak prominent confined to cell cua, dense aggregation of microtrichia around A_1_+Cu_2_; supernumerary lobe weak. ***Abdomen*** (Fig. 96): 2.54 mm long, 2.01 mm wide, oval shaped, tergites free, tergite 1 black, tergite 2 black basally with broad fulvous band, tergites 3–5 black with narrow fulvous markings apically. Tergite 5 with prominent shining spots (ceromata).

***Female genitalia*.** Oviscape dark brown (Fig. 98), conical (1.62 mm), spicules on distal end of eversible membrane (1.42 mm) with six or seven blunt spicules (Fig. 99), aculeus (1.07 mm) with apex trilobed (Fig. 100), spermatheca black, coiled (Fig. 17).

**Figures 102–109.**
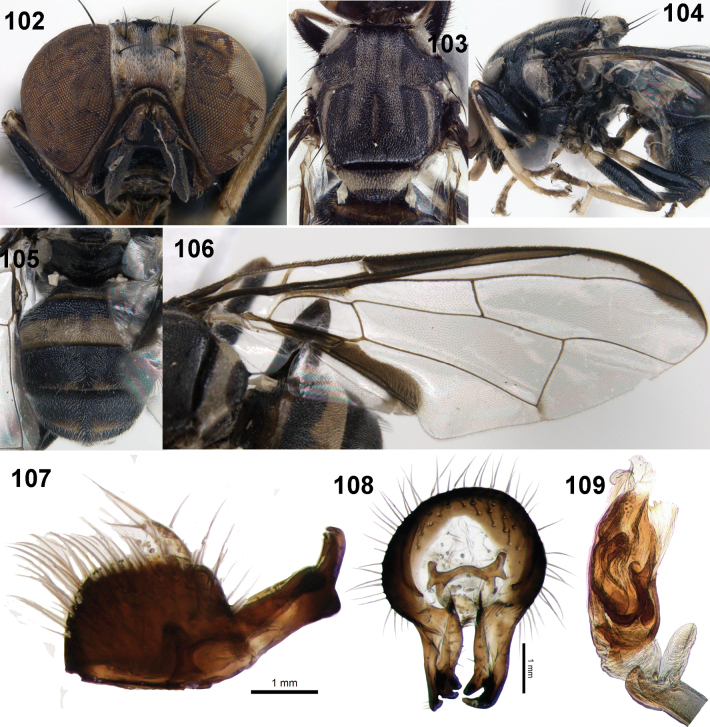
Male of *Zeugodacusumiam* David & Kennedy, sp. nov. **102** head (frontal view) **103** scutum (dorsal view) **104** thorax (lateral view) and legs **105** abdomen **106** wing **107** epandrium and surstyli (lateral view) **108** epandrium and surstyli (posterior view) **109** glans of phallus.

**Male (Figs 102–106).** Similar to female except for entirely black face, broad basal band on the scutellum, dense aggregation of microtrichia around A_1_+Cu_2_, black extensive markings on all femora and pecten on tergite 3.

***Male genitalia*.** Sternite 5 black with shallow emargination, epandrium quadrate with lateral surstylus as long as epandrium, proctiger membranous, not inflated, epandrium sclerotised (Figs 107–108), as long as wide (height 0.31 mm; width 0.28 mm); surstyli slightly shorter than epandrium, oblique, 0.21 mm long; posterior lobe of surstylus 5× longer than anterior lobe, aedeagus 1.93 mm long with glans of phallus (Fig. 109) 0.41 mm long. Three-quarters of glans heavily sclerotised with well-developed patterned acrophallus, praeputium, and subapical lobe.

#### Etymology.

The species name is type locality of the species and is a noun in apposition.

#### Host plant.

Not known.

#### Male parapheromone.

Cue lure.

#### Remarks.

This species is placed in *Zeugodacus* due to shallow/flat posterior emargination of sternite 5 in males, posterior lobe of lateral surstylus much longer than anterior lobe and patterned acrophallus. It is placed in subgenus Zeugodacus as it possesses medial postsutural vitta, postsutural supra-alar and prescutellar acrostichal seta.

##### ﻿New distributional records

### ﻿ Bactrocera (Parazeugodacus) abbreviata

Taxon classificationAnimaliaDipteraTephritidae

(Hardy, 1974)

AAF0DC1F-67EF-5B0D-82F9-B197E6826171

Figs 110–114

Dacus (Zeugodacus) abbreviatus Hardy, 1974: 44.
Bactrocera (Zeugodacus) abbreviata : Norrbom et al. 1999: 101.
Bactrocera (Parazeugodacus) abbreviata (Hardy): Drew and Romig 2016: 243.
Bactrocera abbreviata (Hardy, 1974): Doorenweerd et al. 2018: 23.

#### Material examined.

1♂, India: Meghalaya, Umiam, 06.07.2021. Kennedy N. (NIM).

#### Diagnosis.

(Figs 29–33): This species has been adequately described by Drew and Romig (2013) except for the postabdominal structures. It resembles *B.bipustulata* in possessing scutellum with a medial black band, hyaline wing without costal band, short yellow lateral vitta ending at postsutural supra-alar seta, but can be differentiated by the presence of separate black spots on face, all femora fulvous without fuscous/black markings. Male of the species has been examined for genitalia characters. Epandrium quadrate with proctiger smaller than epandrium; posterior lobe of surstylus as long as anterior lobe, epandrium (oval) in posterior view. Glans of phallus with acrophallus patterned; phallus 1.82 mm. This species was originally described from Philippines, distributed across China and Thailand, is being recorded from Meghalaya, India for the first time.

**Figures 110–114.**
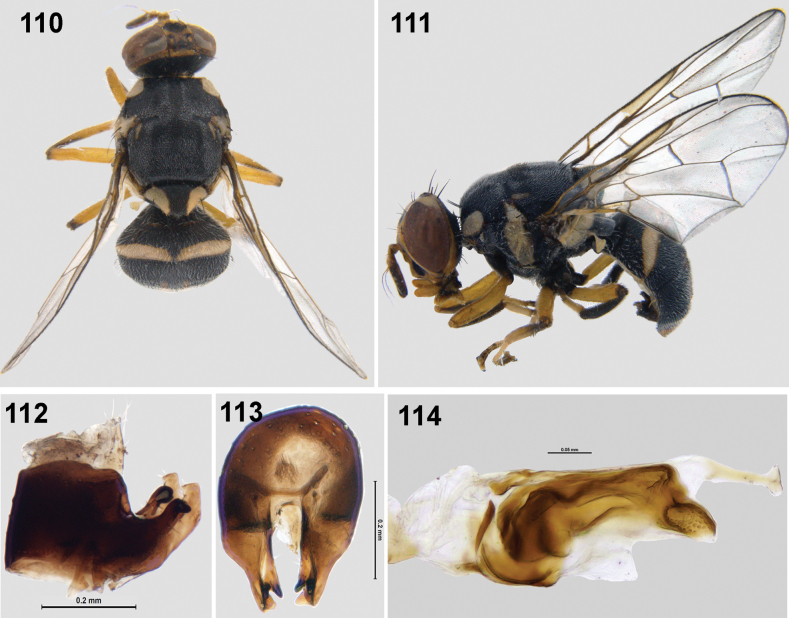
Bactrocera (Parazeugodacus) abbreviata (Hardy) **110** habitus (dorsal view) **111** habitus (lateral view) **112** epandrium and surstyli (lateral view) **113** epandrium and surstyli (posterior view) **114** glans of phallus (lateral view).

#### Male attractant.

Zingerone.

### ﻿ Dacus (Mellesis) vijaysegarani

Taxon classificationAnimaliaDipteraTephritidae

Drew & Hancock, 1998

D56ABC26-1EF4-51D1-973D-1376C45A385B

Figs 115–119

Dacus (Callantra) vijaysegarani : Drew et al. 1998: 636.
Dacus (Mellesis) vijaysegarani : Drew and Romig 2013: 399.
Dacus vijaysegarani : Doorenweerd et al. 2018: 43.

#### Material examined.

1 ♂, India, Meghalaya, Umiam, 29.v.2023, Kennedy N. (NIM).

**Figures 115–119.**
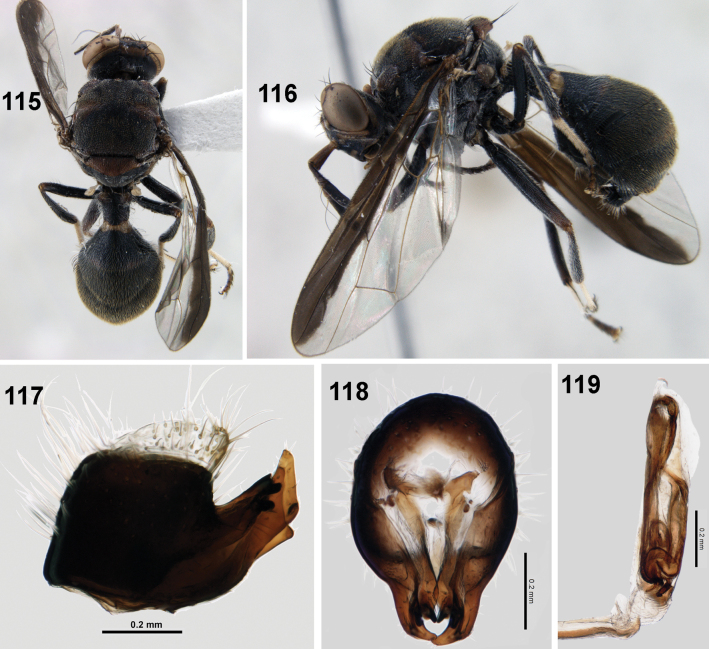
Dacus (Mellesis) vijaysegarani Drew & Hancock **115** habitus (dorsal) **116** habitus (lateral) **117** epandrium (lateral view) **118** epandrium (posterior view) **119** glans of phallus.

#### Diagnosis.

This species has been adequately described by Drew et al. (1998) and Drew and Romig (2013) except for postabdominal structures. A male of the fly collected from Meghalaya was dissected to study the postabdominal structures. Epandrium deeply sclerotised, black, lateral surstylus with posterior lobe slightly longer (2–3×) than anterior lobe; epandrium bulbous (in posterior view), glans of phallus elongate (0.7 mm) with patterned aculeus.

## Supplementary Material

XML Treatment for Zeugodacus

XML Treatment for Zeugodacus (Parasinodacus) momordicae

XML Treatment for Zeugodacus (Zeugodacus) nasivittatus

XML Treatment for Zeugodacus (Sinodacus) sinuvittatus

XML Treatment for Zeugodacus (Zeugodacus) umiam

XML Treatment for Bactrocera (Parazeugodacus) abbreviata

XML Treatment for Dacus (Mellesis) vijaysegarani

## ﻿Additional information

### Author ORCIDs

Karamankodu Jacob David https://orcid.org/0000-0002-5092-141X

Venkateshaiah Abhishek https://orcid.org/0009-0002-7044-3977

Ningthoujam Kennedy https://orcid.org/0000-0002-6709-7252

K. M. Ajaykumara https://orcid.org/0000-0002-4553-3068

R. G. Gracy https://orcid.org/0000-0002-6764-5167

Cheday Bhutia Hissay https://orcid.org/0009-0000-2819-8088

### Data availability

All of the data that support the findings of this study are available in the main text.
